# RNA-Sequencing for profiling goat milk transcriptome in colostrum and mature milk

**DOI:** 10.1186/s12917-016-0881-7

**Published:** 2016-11-25

**Authors:** Alessandra Crisà, Fabrizio Ferrè, Giovanni Chillemi, Bianca Moioli

**Affiliations:** 1 Consiglio per la ricerca in agricoltura e l’analisi dell’economia agraria (CREA) - Animal production research centre, Via Salaria 31, 00015 Monterotondo, Rome Italy; 2 Department of Pharmacy and Biotechnology (FaBiT), University of Bologna Alma Mater, Via Belmeloro 6, 40126 Bologna, Italy; 3 Applications and Innovation Department, CINECA, SCAI SuperComputing, Via dei Tizii 6, 00185 Rome, Italy

**Keywords:** Goat milk, Transcriptomic profile, RNA-seq, Lactation, Colostrum, Oligosaccharides, Milk somatic cells

## Abstract

**Background:**

In this work we aimed at sequencing and assembling the goat milk transcriptome corresponding at colostrum and 120 days of lactation. To reconstruct transcripts we used both the genome as reference, and a *de novo* assembly approach. Additionally, we aimed at identifying the differentially expressed genes (DEGs) between the two lactation stages and at analyzing the expression of genes involved in oligosaccharides metabolism.

**Results:**

A total of 44,635 different transcripts, organized in 33,757 tentative genes, were obtained using the goat genome as reference. A significant sequence similarity match was found for 40,353 transcripts (90%) against the NCBI NT and for 35,701 (80%) against the NR databases. 68% and 69% of the *de novo* assembled transcripts, in colostrum and 120 days of lactation samples respectively, have a significant match with the merged transcriptome obtained using Cufflinks/Cuffmerge. *CSN2, PAEP, CSN1S2, CSN3, LALBA, TPT1, FTH1, M-SAA3, SPP1, GLYCAM1, EEF1A1, CTSD, FASN, RPS29, CSN1S1, KRT19* and *CHEK1* were found between the top fifteen highly expressed genes. 418 loci were differentially expressed between lactation stages, among which 207 and 122 were significantly up- and down-regulated in colostrum, respectively. Functional annotation and pathway enrichment analysis showed that in goat colostrum somatic cells predominate biological processes involved in glycolysis, carbohydrate metabolism, defense response, cytokine activity, regulation of cell proliferation and cell death, vasculature development, while in mature milk, biological process associated with positive regulation of lymphocyte activation and anatomical structure morphogenesis are enriched. The analysis of 144 different oligosaccharide metabolism-related genes showed that most of these (64%) were more expressed in colostrum than in mature milk, with eight expressed at very high levels (*SLCA3, GMSD, NME2, SLC2A1, B4GALT1, B3GNT2, NANS, HEXB*).

**Conclusions:**

To our knowledge, this is the first study comparing goat transcriptome of two lactation stages: colostrum and 120 days. Our findings suggest putative differences of expression between stages and can be envisioned as a base for further research in the topic. Moreover because a higher expression of genes involved in immune defense response, carbohydrate metabolism and related to oligosaccharide metabolism was identified in colostrum we here corroborate the potential of goat milk as a natural source of lactose-derived oligosaccharides and for the development of functional foods.

**Electronic supplementary material:**

The online version of this article (doi:10.1186/s12917-016-0881-7) contains supplementary material, which is available to authorized users.

## Background

One of the most important livestock and the oldest domesticated species raised worldwide is the goat (*Capra hircus*). Goats have long been used for their milk, meat, hair (including cashmere), and skins throughout much of the world and this species is also one of the best model organisms for mammary gland bioreactor studies [1]. Today, there are > 1000 goat breeds, and > 900 million goats are kept around the world according to a report by the UN Food and Agriculture Organization [2, 3]. Despite the agricultural and biological importance of goats, breeding and genetics studies have been delayed due to the lack of a reference genome. In [4] it was reported for the first time the sequencing and automated whole-genome optical mapping of the goat genome. Besides its nature of complete food able to provide nutritional subsistence of infants, healthy growth of children and nourishment of adult humans, milk contains a plethora of components that encode functional health benefits to consumers [5]. Among dairy livestock, goats produce milk containing free milk oligosaccharides (OS) at much higher levels than cattle or sheep [6, 7] and different studies have shown that their concentration is higher in colostrum than in the subsequent lactation stages [8–10]. A vast number of functional in vitro and in vivo studies gave evidence of the biological functions of OS in human health and nutrition [11–15]. Moreover, goat milk contains a significant variability of OS, similar to the one observed in human milk, so to hold high potential as a natural source of lactose-derived OS as supplement for infant formulae and for the development of functional foods, having been successfully isolated by membrane technology [7, 16]. Goat milk contains both neutral and acidic OS. Galactose (Gal), glucose (Glc), fucose (Fuc), Nacetylglucosamine (GlcNAc) and N-acetylgalactosamine (GalNAc) grafted on a lactose core make up neutral OS; these same monomers, plus N-acetylneuraminic acid (NeuAc) and N-glycolylneuraminic acid (NeuGc), comprise acidic OS [17, 18]. Oligosaccharides chains covalently linked to glycoprotein and glycolipids often confer specific biological functions to the molecules carrying them such as signaling, adherence and cellular migration through the body. Glycosylation of proteins and lipids plays a crucial role in numerous biological processes including the regulation of immune and inflammatory responses [19]. To understand the function of glycoconjugated it is important to investigate the regulation of expression of the genes encoding the “glycosylation-related” genes, such as the large families of glycosyltransferase and glycosidase [20–22].

Milk component synthesis and secretion by the mammary gland involve expression of a large number of genes. Formerly, many studies of gene expression were confined to the analysis of a limited number of selected genes of interest, e.g. the genes encoding the key lipogenic enzymes in the mammary gland [23, 24]. The recent development of Next-Generation Sequencing technologies allows a much broader and detailed knowledge on the biology of the mammary gland [25]. RNA-seq transcriptomic analysis has been widely used for model and non-model organisms and in particular for organisms for which limited genomic resources are available [26–29].

Principal aim of the present study aim was to profile the transcriptome of goat milk somatic cells (GMSCs) by using an RNA-seq approach. Because, currently, there are no official gene annotations for the goat genome, we decided to employ two strategies to reconstruct transcripts as accurate as possible: 1. using the genome as reference to build transcripts from the sequencing reads, and 2. using a *de novo*, genome-independent assembly approach. Additionally, we aimed to identify the differentially expressed genes (DEGs) between two lactation stages, colostrum (D1) and 120 days (D2); in fact, colostrum composition highly differs from milk composition once the lactation is established, being the colostrum richer in nutrients and characterized by high levels of bioactive components [30]. Finally, we also aimed to compare, in the two different lactation stages, the expression of genes involved in OS metabolism, which, to date, was never analyzed in goats, but calls upon utmost interest, as shown by the studies on the expression of glycosylation-related genes, in mouse [31], humans [20, 32] and cattle [33].

## Methods

### Sample collection and RNA extraction

RNA used in the present trial was obtained from goat milk somatic cells (GMSCs), in agreement with [34], who showed that GMSCs can be used to accurately reveal the cellular dynamics of mammary gland gene expression, and with other authors [35–37] who demonstrated an extensive similarity between the mammary gland and MSC/mammary epithelial cells transcriptome.

Milk was hand collected (both udder halves) from a Maltese goat, first parity, raised in a farm privately owned and traditionally managed. Two lactation stages were sampled: colostrum (D1) and 120 days milk (D2). Fifty mL of milk were transferred to falcon tubes and immediately centrifuged at 2000 g for 10 min at 20 °C in the presence of 50 μl EDTA 0,5M pH 8. The fat layer on the top of the supernatant was removed with a sterile pipette tip and the skim milk was discarded. The milk somatic cell-pellets were resuspended in 1 ml PBS 1× with 300 μl EDTA 0,5M pH 8. After centrifugation the pellets were lysed with 1 mL TRI REAGENT (Sigma-Aldrich, Milan, Italy) and total RNA was extracted following the TRI REAGENT protocol. Samples were digested with RNase-Free DNase Set (Qiagen, Milan, Italy) to remove DNA contamination and finally cleaned with RNeasy Minelute kit (Qiagen, Milan, Italy). RNA quality and quantity was checked with spectrophotometric (NanoPhotometer™ Pearl, Implen GmbH, München, Germany) and microfluidic inspections (RNA 6000 Nano chip, 2100 Bioanalyzer, Agilent Technologies, Milan, Italy).

### Illumina sequencing and transcriptome assembly

The RNA was sequenced by using the ARK-Genomic center service at The Roslin Institute, University of Edinburgh [38]. Samples taken on for processing had an RNA integrity number (RIN) value greater than 7. Briefly, the polyadenylated RNA was isolated onto beads to remove it from all of the ribosomal RNAs, was then fragmented to an average size of 180 bases and reverse-transcribed to cDNA using random oligonucleotide primers. Following second-strand cDNA synthesis, end repair, and addition of a single A base, Illumina RNA-seq primers were ligated to the A-tailed double stranded cDNA. The ligated adapters were extended by PCR to add the sample barcode sequences and the resulting PCR products were assessed for quality using the Agilent Bioanalyzer DNA 1000 chip (average insert length of 175 bases) and for quantity using the Kapa Biosciences library quantification kit (Illumina). Following single stranding, dilution, loading onto an Illumina V2 flow cell for the Genome Analyzer IIx instrument, using a cBot following the manufacturer’s recommendations, the samples were finally sequenced for 79 bases paired end in a single lane of an Illumina Genome Analyzer IIx. The data was extracted to FastQ format using the Casava software from Illumina.

The raw read data from this study were deposited to the NCBI Sequence Read Archive (SRA) under accession number SRA261702. Read quality was examined using FastQC (v0.10.1) [39]. After adapter sequence removal, read ends were dynamically trimmed if base quality scores were lower than Q20 using Trimmomatic [40]. At the end of this process, reads were discarded if the total length of the read was shorter than 10 bases or if the fraction of undetermined bases was >2%.

The clean reads were subsequently analyzed with two approaches: a) by using the Tuxedo Suite, comprising of Bowtie (v2.1.0) [41], TopHat (v2.0.9) [42] and Cufflinks (v2.1.1) [43]. In particular, we aligned the sequence reads to the reference goat genome assembly AJPT0000000.1 [4] with a tolerance of two mismatches (Bowtie), we identified splicing junctions (TopHat), and we reconstructed transcripts and measured their expression levels (Cufflinks) reported as Fragments Per Kilobase of transcript per Million mapped reads (FPKM). b) by using the Inchworm RNA-Seq Assembler in the Trinity software [44], we performed a *de novo* transcriptome assembly, and we mapped the resulting contigs (corresponding to transcripts) to the goat genome using Gmap [45]. Additionally, Cufflinks was run in two different ways: a) using the available goat transcript sequences from the RefSeq database; b) without transcript information. For the latter, the Cufflinks generated transcriptomes obtained independently for the D1 and the D2 sample were merged using Cuffmerge, part of the Cufflinks distribution, to obtain a reference transcriptome. For the former, we started from the 27,946 RefSeq goat transcripts (as of July 2015), of which 27,649 reported as predicted, then we determined their genomic coordinates by using BLAT [46], converted them into General Feature Format (GFF), and feed this GFF file to Cufflinks to compute expression values, in FPKM units, using the Bowtie/TopHat mapped reads for the two samples.

Transcripts generated by Trinity and Cufflinks were compared to each other using BLASTN, requiring a significant match (E-value < = 10E-5) covering at least 50% of the longest transcript sequence.

Venn diagrams were obtained by using eulerAPE software [47].

### Functional annotation and pathway enrichment analysis

The Cufflinks/Cuffmerge and the Trinity transcripts were BLASTed against the NCBI GenBank nucleotide (NT) or non-redundant protein (NR) databases to annotate them. A match was accepted if the BLAST top hit E-value was smaller than 10E-5. Transcripts for which no sufficiently good matches were found after this procedure were classified as ‘unannotated’. Gene symbols were associated to each match using bioDBNet [48]. Gene ontology (GO) and KEGG pathways annotations were obtained using Blast2GO [49] against the NR database (E-value cutoff 10E-5). This method has been applied to the full set of transcripts obtained for the two samples.

Lists of highly expressed genes and lists of differentially expressed genes (DEGs) between stages were characterized using the free Database for Annotation, Visualization and Integrated Discovery (DAVID) v6.7 web-service [50, 51]. Enrichment in the GO three ontologies and in pathways was evaluated by DAVID by means of a modified Fisher Exact Test (EASE), using an EASE score threshold of 0.1. The DAVID Functional Annotation Clustering tool was used to group annotations, setting stringency to Medium and using default values for the other parameters. The Enrichment Score was used to rank functional clusters, and a representative annotation was chosen to represent each group as the one having lowest enrichment *p*-value. Clusters having no term with *p*-value < 0.05 were discarded.

### Differential gene expression profiling

NOISeq was used to evaluate differential gene transcription between stages [52]. Read counts for each gene were compiled using HTSeq [53], normalized using the tmm (trimmed mean of M values) method, and analyzed by NOISeq using parameters nss = 5 (number of simulated replicates), pnr = 0.2 (size of simulated samples), v = 0.02 (simulated samples variability). Differentially expressed genes (DEGs) where selected as those with probability q > = 0.9. The WEB-based GEne SeT AnaLysis Toolkit (WebGestalt) software was used for functional enrichment analysis of the differential expressed genes [54, 55]. This tool integrates functional categories derived from centrally and publicly curated databases as well as computational analyses; it adds functional categories defined by hierarchical protein interaction network modules. The default software options were used with Benjamini-Hochberg multiple test adjustment. The WebGestalt software resulted in the visualization feature DAGs (directly acyclic graph) that not only revealed the hierarchical relationship of enriched GO terms but also extended to define relationship among genes in a network module. For the pathway collection, in addition to the KEGG database, WebGestalt has added data from Pathway Commons, which collects and integrates nine centrally curated biological pathway database, and WikiPathways, that is a primary source for open community-based curation. The Search Tool for the Retrieval of Interacting Genes/Proteins (STRING v9.1) database and web tool [56] was used to provide a critical assessment and integration of protein-protein interactions, including direct (physical) as well indirect (functional) associations. Interactions are derived from multiple sources. The probabilistic confidence score that is assigned to associations, is derived by separately benchmarking groups of associations against the manually curated functional classification scheme of the KEGG database. The various major sources of interaction/association data in STRING are benchmarked independently; a combined score is computed which indicates higher confidence when more than one type of information supports a given association. The “minimum required interaction score” puts a threshold on the confidence score, such that only interactions above this score are included in the predicted network. Lower thresholds lead to more interactions, but also to more false positives. The confidence score is the approximate probability that a predicted link exists between two enzymes in the same metabolic map in the KEGG database. For the present study, we considered *Bos Taurus* as the reference organism and a minimum required interaction score of 0.7, which corresponds to a high-confidence predicted network.

### Enrichment of sugar metabolism genes

We edited a list of genes implicated in milk OS metabolism and of genes relative to the lactose synthesis pathway following [33] and [57] respectively, with integrations. A list of 144 genes belonging to different functional OS metabolism categories was examined in the milk goat transcriptome. Goat orthologs were identified by using BLASTX against the NCBI NR (E-value < 10E-5), and expression values of the orthologs were computed by Cufflinks, as described above.

## Results

### RNA-seq mapping statistics and *de novo* assembly

RNA sequencing produced a total of ~ 100 million reads per sample; the number of reads obtained before and after the quality filtering are reported in Table 1. The raw read data were deposited to the NCBI Sequence Read Archive (SRA) under accession number SRP057582.

**Table 1:** Summary of read counts and alignments

Samples	Total reads	Clean reads	Mapped reads	Mapped/clean
D1	115,900,892	115,402,457	106,631,870	92.4%
D2	109,955,930	109,511,963	100,312,958	91.6%

Two strategies were employed to profile the goat milk transcriptome to overcome the limits of the currently available goat genome sequence and transcriptome annotations. We employed the putative transcripts provided by RefSeq through the Bowtie/TopHat/Cufflinks pipeline, but we also let the software reconstruct genes and transcripts directly from the read mapping. These pipelines were applied independently to both lactation samples, and the resulting transcripts were then merged and annotated. We also reconstructed the transcriptome using a *de novo* approach (i.e. without using a reference genome) by means of the software Trinity, with the aim of detecting transcripts encoded in missing or mis-assembled loci of the current genome assembly. Results of both approaches were compared and finally integrated.

The Cufflinks procedure when the RefSeq annotations were not provided generated 37,969 transcripts for the D1 sample, having an average length of 1,947 bp, organized in 31,311 putative genes, while the D2 sample generated 38,533 transcripts from 31,817 putative genes, 1,896 bp long on average. A total of 44,635 different transcripts, having average length of 2,296 bp (the longest transcript is 33,250 bp long) was obtained by merging the two datasets. The merging procedure, using Cuffmerge, also automatically discarded potential artifacts. These transcripts are organized in 33,757 clusters (i.e. tentative genes), which encode for 1.3 splicing variants on average (Additional file 1). All these transcripts were BLASTed against the NCBI nucleotide (NT) and non-redundant protein (NR) databases; a significant match was found for 40,353 of them (90%) against the NT and for 35,701 (80%) against the NR databases (Table 2).

**Table 2:** Statistics of the Cufflinks transcripts

Samples	Transcripts (Cufflinks)	Putative genes (Cufflinks)	Averaged fragments length (Cufflinks)	Total merged transcripts (Cuff merge)
D1	37,969	31,311	1,947	44,635 of which 40,353 (90% match against NT) 35,701 (80% match against NR)
D2	38,533	31,817	1,896	

The *de novo* transcriptome assembly for the D1 sample produced 136,420 assembled fragments from 75,147 clusters (i.e. putative genes), with 1,311 bp of fragment average length, of which 112,001 (82%) map to the goat genome assembly with at least 90% identity and 90% sequence coverage. 58,495 fragments (43%) found a significant match with the NCBI NR and only 5404 assembled fragments (0.03%) do not show any significant match with the assembly. A total of 142,344 fragments from 73,823 putative genes (average fragment length 1,363 bp) were assembled from the D2 sample reads; 115,686 fragments (82%) map well on the goat genome (only 0.05% do not have any significant genomic match), while 66,919 (47%) have a significant hit in the NCBI NR (Table 3).

**Table 3:** Statistics of the *de novo* transcripts

Samples	Transcripts *de novo*	Averaged fragments length *de novo*	Transcripts mapped to goat genome (90% identity)	Transcripts matched with BLAST NR (E-value 10E-5)
D1	136,420	1,311	112,001 (82%)	58,495 (43%)
D2	142,344	1,363	115,686 (82%)	66,919 (47%)

**Fig. 1:**
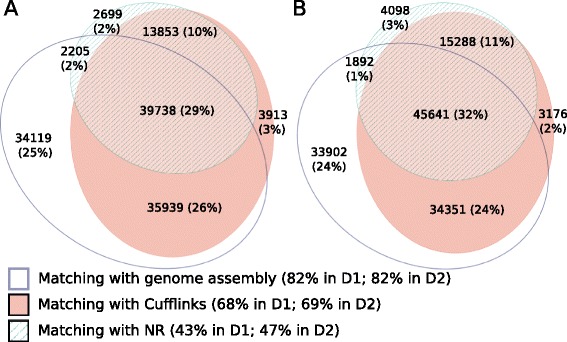
Matches between the *de novo* transcripts with goat genome, Cufflinks/Cuffmerge transcripts, NR database. **a** *De novo* D1 transcripts match with goat genome, Cufflinks/Cuffmerge transcripts and NR database. **b** *De novo* D2 transcripts match with goat genome, Cufflinks/Cuffmerge transcripts and NR database

The *de novo* assembled transcripts in the two lactation stages show a relevant correspondence with the merged transcriptome obtained using Cufflinks/Cuffmerge (44,635), as detected by BLASTing the former against the latter: 68% of the D1 sample *de novo* transcripts, and 69% of the D2 sample transcripts, have a significant match (E-value < 10E-5), and for 55% of both the D1 and the D2 sample transcripts, the match also covers at least 50% of the transcript sequence. While the assembled transcripts align remarkably well with the goat genome assembly, they must be taken cautiously since a large fraction of them is likely incomplete (i.e. not full length transcripts). To further validate them, we compared the *de novo* transcripts with those reconstructed by the Cufflinks/Cuffmerge pipeline, discarded those that find a good correspondence, and selected for further analysis those that, while not having a match with the genome, found a significant match in the NCBI NR database. The rationale is that this subset of *de novo* transcripts are more likely encoded by regions not present or mis-assembled in the current goat genome assembly and therefore missed by Cufflinks/Cuffmerge. Figure 1a reports as a Venn diagram a classification of the D1 *de novo* transcripts, that shows how many have a significant match with those reconstructed using the goat genome as reference by the Cufflinks pipeline, how many have a significant match with the NR database, and how many align well on the genome assembly, and the overlap between these groups. For example, a relevant portion of all transcripts (39,738, 29% of the total) shows significant matches with the Cufflinks transcripts, the NR and the genome, and corresponds to the region of overlap of the three groups at the center of the Venn diagram; another large group (35,939, 26%) aligns well on the genome and with the Cufflinks transcripts, but not with the NR, and corresponds to the overlap region between these two groups at the bottom of the Venn diagram. Figure 1b reports a similar Venn diagram for the D2 sample *de novo* transcripts. In this case, transcripts that have significant alignment with the Cufflinks transcripts, the NR and the genome are 45,641 (32% of the total), while those having a good match with the Cufflinks and the genome but not with the NR are 34,351 (24%). These might represent goat-specific transcripts, even if a fraction might not find a significant match in the NR because their sequence was not fully reconstructed, or reconstructed only or mainly in a non-translated region, or by the lack of an homologous protein in the database. In general, there is a good agreement between transcripts reconstructed using the genome as reference and those reconstructed using a *de novo* approach, and many of them can be annotated using a match with the NR dataset; this holds true for both the D1 and the D2 sample, and the relative size of these groups of transcripts is similar in the two samples. Yet, a large fraction of the *de novo* transcripts cannot be annotated.

A small fraction of transcripts (2699 for the D1 sample and 4098 for the D2 sample) find a significant match in the NR while not having correspondence either with the goat genome or with the Cufflinks transcripts. We suggest that those transcripts are encoded by loci not present or not well assembled in the current goat genome assembly. On the other hand, many *de novo* transcripts do not show any significant match with the Cufflinks/Cuffmerge transcripts, even though they can be mapped reliably to the goat genome assembly. Specifically, these are 36,324 for the D1 sample (the 34,119 that match only with the genome assembly plus the 2,205 that match with both the genome and the NR) and 35,794 for the D2 sample (33,902 plus 1,892), as shown in Fig. 1. Nevertheless, in the majority of cases (30,556 for the D1 and 30,672 for the D2 sample), even if the sequence of these transcript cannot be aligned significantly with any Cufflinks transcript, the mapped genomic coordinates of these *de novo* transcripts overlap with one of the loci reconstructed by Cufflinks with at least 200bp, suggesting that these transcripts are transcribed by the same loci. The lack of significant similarity as detected by BLASTing can be ascribed to errors in the transcript assembly, to the ability of Trinity and Cufflinks in reconstructing different portions of the transcript, or to incorrect reconstruction of alternative splicing events. The 3553 remaining *de novo* D1 and 3997 D2 sample transcripts need further investigation. A relevant fraction of the highly expressed transcripts (182 out of 798, 23% in the D1 sample; 180 out of 644, 28% in the D2 sample) did not show any significant match with the NCBI NR dataset. These un-annotated transcripts tend to be short, and nucleotide BLAST versus the NCBI NT collection showed that most of them find significant matches with ribosomal RNAs, suggesting that they might result from rRNA sample contamination.

### Milk gene expression statistics

We first analyzed expression data, considering the transcripts obtained with the Cufflink procedure, setting four thresholds for the FPKM values (0.1, 1, 5, 15). Thereafter divided them into high (> = 500 FPKM), medium (> = 10 to 500 FPKM), and low expression (<10 FPKM) (Table 4). We observed a general agreement between the two samples (Table 4), both when using and when not using RefSeq predicted transcript annotations. In particular, there were 798 (3%) and 644 (2%) highly expressed genes in the D1 and in the D2 sample respectively when the Cufflinks procedure was employed without providing the RefSeq transcript coordinates, of which 496 genes are common between them. In general, gene expression resulted similar in the two considered samples, regardless of the analysis method.

**Table 4:** Gene expressed in D1 and D2 or co-expressed (i.e. found expressed in both samples) as percentage of the total number of transcripts and following the categorization

	Without annotations			With RefSeq annotations		
Category	D1	D2	Co-expressed	D1	D2	Co-expressed
FPKM value > =0.1	31,040 (95%)	31,136 (95%)	29,392	16,646 (60%)	16,200 (58%)	15,242
FPKM value > =1	17,188 (52%)	17,751 (54%)	15,768	11,327 (41%)	10,634 (38%)	10,203
FPKM value > =5	8,800 (27%)	8,472 (26%)	7,801	6,028 (22%)	4,983 (18%)	4,826
FPKM value > =15	4,465 (14%)	3,876 (12%)	3,643	3,017 (11%)	2,153 (8%)	2,095
Highly expressed genes (> = 500 FPKM)	798 (3%)	644 (2%)	594	164 (0.06%)	104 (0.03%)	103
Medium expressed genes (> = 10 FPKM to 500 FPKM)	7,733 (24%)	7,771 (24%)	6,856	3,782 (14%)	2,888 (11%)	2,750
Lowly expressed genes (<10FPKM)	24,453 (74%)	24,512 (75%)	23,663	23,460 (86%)	24,414 (89%)	23,383

**Fig. 2:**
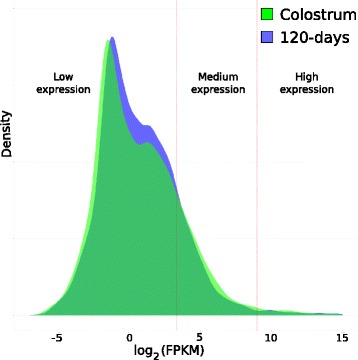
Plot of the log2 (FPKM) value distribution in the D1 and D2 samples. Vertical *red* lines show the gene expression groups

In order to further categorize the genes with different level of expression, a multiphasic graph was obtained by plotting the log2 transformed FPKM value versus the expressed genes (Fig. 2). Two peaks, at 0.2 and 1 FPKM, respectively, are visible.

### Global milk transcriptome functional annotation and pathway analysis

To look at the different pattern of biological functions in the two studied lactation stages, transcripts reconstructed by Cufflinks in the D1 (31,311) and D2 sample (31,817) were annotated independently using the Blast2GO pipeline, starting from the BLASTX against the NR results. A total of 14,179 transcripts from the D1 and 14,620 from the D2 sample can be associated with at least one GO term. Distribution of GO terms and KEGG pathways were investigated, resulting quite similar. Both D1 and D2 sample GO terms lists, ranked by the Blast2GO score show at the top rather generic GO terms related to transcription regulation, signal transduction, transport, metabolic processes, cell division, phosphorylation, and others. It is to be expected that these two samples, that share the expression of the large majority of genes, also show similar GO terms distributions.

**Fig. 3:**
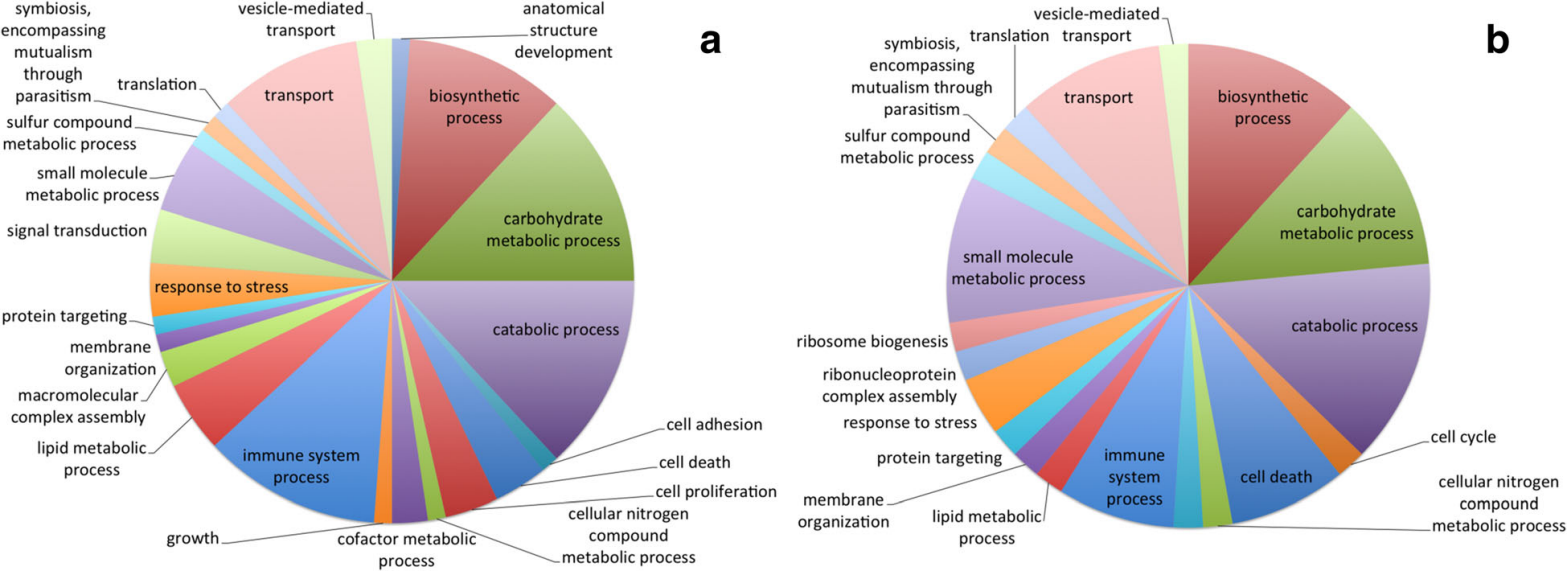
Pie charts of the GO terms associated with the expressed genes in the two analyzed samples, D1 (**a**) and D2 (**b**). Each GO term detected by blast2go on the results of the BLAST against the NCBI NR database was mapped to its corresponding GOSlim (a selection of representative GO terms maintained by the Gene Ontology Consortium)

We also assessed the enrichment in the number of genes associated to a specific GO term or pathway comparing one sample to the other (Fisher exact test, *p*-value < = 0.05, FDR 5%). In the D1 sample, there was enrichment in 67 terms associated to cellular metabolic processes, cellular organization, biosynthesis, and response to stimuli (Additional file 2 and Fig. 3a). In the D2 sample, instead, the 20 enriched GO terms included several terms related to apoptosis (positive regulation of cell death, positive regulation of apoptotic process, induction of programmed cell death, developmental programmed cell death) (Additional file 2 and Fig. 3b). The examination of KEGG pathway categories and subcategories indicated that a large number of pathways (124) results expressed in both samples, and the most represented ones are related to purine and pyrimidine metabolism, aminoacyl-tRNA biosynthesis, inositol and phosphatidylinositol metabolism and signaling, glycerolipid, glycerophospholipid and sphingolipid metabolism, lysine degradation, oxidative phosphorylation, amino- and nucleotide-sugar metabolism, and T cell receptor signaling. Three pathways (amino sugar and nucleotide sugar metabolism, lysine degradation, steroid hormone biosynthesis) are enriched in the D1 with respect to the D2 sample in terms of number of expressed pathway members (Fisher exact test, *p*-value < = 0.05, FDR 5%). Two pathways, the naphthalene degradation and the lipoic acid metabolism pathways, are specific for the D1 sample, even though represented by very few expressed genes, while no pathway appears specific for the D2 sample (Additional file 3).

### Top genes expressed in colostrum and mid lactation

In general the dynamic range of gene expression intensities covered several orders of magnitude and gene expression values in the two samples were highly correlated (Pearson correlation coefficient 0.93). The fifteen genes with the highest FPKM values in D1 and/or D2 sample were β-casein (*CSN2*), progestagen-associated endometrial protein (*PAEP*), α-S2 casein (*CSN1S2*), κ-casein (*CSN3*), α-lactalbumin (*LALBA*), tumor protein translationally-controlled 1 (*TPT1*), ferritin heavy chain 1 (*FTH1*), serum amyloid A3 (*M-SAA3*, goat locus LOC100860781), secreted phosphoprotein 1 (*SPP1*), translationally-controlled tumor protein-like (*LOC102179679*), glycosylation dependent cell adhesion molecule-1 (*GLYCAM1*), eukaryotic translation elongation factor 1 alpha 1 (*EEF1A1*), cathepsin D (*CTSD*), fatty acid synthase (FASN), ribosomal protein S29 (*RPS29*), α-S1 casein (*CSN1S1*), keratin 19 (*KRT19*) and checkpoint kinase 1 (*CHEK1* or *CHK1*) (Table 5).

**Table 5:** List of the fifteen highly expressed genes (in bold) in the D1 and D2 samples, with their respective FPKM values

Gene symbol	D1 (FPKM)	D2 (FPKM)
*CSN2*	**121511**	**320950**
*PAEP or BLG*	**65569**	**45561**
*CSN1S2*	**48645**	**103105**
*CSN3*	**47454**	**111404**
*LALBA*	**39379**	**60454**
*TPT1*	**12008**	**7603**
*FTH1*	**11827**	**6563**
*M-SAA3*	**10640**	3141
*SPP1*	**10047**	**7286**
*LOC102179679*	**7751**	**3769**
*GLYCAM1*	**7034**	**18498**
*EEF1A1*	**6294**	**3463**
*CTSD*	**5532**	2974
*FASN*	**4995**	**3580**
*RPS29*	**4775**	1949
*CSN1S1*	2113	**5089**
*KRT19*	4103	**4806**
*CHEK1*	2325	**3923**

Ten of the fifteen highly expressed genes in D1 (*PAEP, TPT1, FTH1, M-SAA3, SPP1, LOC102179679, EEF1A1, CTSD, FASN, RPS29*) showed a decrease in expression in the D2 sample, while five (*CSN2, CSN1S2, CSN3, LALBA, GLYCAM1*) showed a gene expression increase from D1 to D2 lactation stages. In D2 sample three genes, *CSN1S1, KRT19, CHEK1*, showed a higher expression relative to D1 sample. Only three genes out of fifteen (*M-SAA3, CTSD, RPS29)* are highly expressed in one condition but not in the other.

### Functional annotation analysis of highly expressed genes during lactation

To better characterize only the most abundantly transcribed genes, the groups of highly expressed genes described before (FPKM ≥ 500) in the two lactation stages were subjected to gene ontology classification and functional analysis using DAVID (Additional file 4). Annotations results showed more significant terms in the three GO categories and in the annotation cluster for D1 sample than for D2. Overall, the data indicated that in both samples highly expressed genes are involved in mRNA translation, protein localization, transport and modification, ATP biosynthesis, regulation of apoptosis and cell proliferation. In the GO apoptosis relative terms, some gene were ubiquitously expressed both with positive and negative regulation action (*HMOX1, GRX1, UBB, UBA52, RPS27A, CIDEA, NOTCH1, CDKN1A, SQSTM1*), some were implicated in the positive regulation (*LALBA, TXNIP, PRDX1, RPS3, CD44, JUN, RPL11*) and some in the negative regulation (*CCL2, NFKBIA, TPT1, HSPA5, GSTP1*).

Both in colostrum and in late lactation we found statistically significant (*P* < 0.001) enrichment of genes associated with GO terms related to nervous system (long-term strengthening of neuromuscular junction, axonogenesis, regulation of transmission of nerve impulse).

In the D1 sample most genes were specifically involved in carbohydrates metabolism (e.g. glucose and hexose catabolism), oxidative phosphorylation, regulation of unfolded protein, defense response, cellular homeostasis. We identified the solute carrier family 2 (facilitated glucose transporter), member 3, gene (*SLC2A3*) highly expressed in both lactation stages.

Heat shock protein genes (*HSPA5, HSPA6, HSPA8, HSP90AA1, HSP 70.1*) were found between the highly expressed genes with *HSPA1A* and *HSPB1* identified between the differentially expressed genes up-regulated in colostrum. GO-BP classification for *HSP* genes showed association with the following terms: response to unfolded protein, negative regulation of programmed cell death, negative regulation of apoptosis, response to extracellular stimulus, chaperone mediated protein folding requiring cofactor, response to endoplasmic reticulum stress, ‘*de novo*’ posttranslational protein folding, cellular response to stress, negative regulation of peptidase activity.

The highly expressed genes in D2 sample included genes involved in positive regulation of apoptosis, in hydrogen peroxide catabolic process, in regulation of protein transport, modification process and localization, in negative regulation of response to stimulus and of cytokine secretion.

### Differentially expressed genes between lactation stages

Differentially expressed loci (DEGs) between the D1 and the D2 samples were identified using expression levels of the 33,757 transcripts reconstructed by the Cufflinks/Cuffmerge procedure and the goat genome assembly as reference. A total of 418 transcripts, of which 401 were annotated to 329 distinct gene symbols, have NOISeq differential expression probability q > = 0.9. Of these, 275 reconstructed transcripts associated to 207 different gene symbols were up-regulated in D1, and 143 transcripts associated to 122 different genes were down-regulated (Additional file 5). The DAVID and WebGestalt tools were used for the functional enrichment analysis of these two gene lists. These web-services do not support all the organisms and gene identifiers, so we set the *Homo sapiens* species as reference. 169 out of 207 and 111 out of 122 were the up and down regulated genes in D1 sample included in the final analyses. Most GO categories in which DEGs were annotated correspond to those already described for the highly expressed genes.

### Functional enrichment analysis of DEGs in D1 sample

The functional characterization of the annotated up-regulated genes in D1 showed enriched genes associated with GO-BP categories involved in glycolysis, carbohydrate metabolism and catabolism, defense response, cytokine activity, regulation of growth, cell death, vasculature development. The most important GO-MF terms were associated with protein binding and chemokine activity and the most important GO-CC terms were associated with cytoplasm and extracellular space (Additional files 6, 7).

Between the DEGs up-regulated in D1 sample we found the carbohydrate transporter encoding genes *SLC2A1* and *SLC2A10* and the nucleotide sugar transporter gene *SLC35E1*.

Higher expression of genes annotated in immune and defense terms *(IL1R2, CCL3, CCL2, OLR1, C3, CLU, CCL5, CCL4, FCRL4, CCR9, GPI, BPI, PPBP, FCAR, CCL20, SEMA3C, IL12B, LBP, CFI, NFIL3, PTX3, DMBT1)* was also here identified.

Many DEGs up-regulated in D1 were found in GO terms relative to positive (*CCL2, PRTN3, CSF1, CLU, IGF1, ADM, F3, JUN, TGIF1, IL12B, FGF1, IL13RA1, SCG2*) and negative (*INHBA, ACVR1B, CDA, ING1, ENO1*) regulation of cell proliferation and cell growth. Otherwise other genes were identified in terms relative to apoptosis and programmed cell death (*FASTKD3, DLC1, EGLN3, DDIT4, MAGED1, ECE1, JUN, SIAH2, PPP1R15A, FGF2, PHLDA1, SOX9, PPIF, INHBA, ACVR1B, F3, IL12B, IGFBP3*) with some as negative regulators (*NOL3, CCL2, BAG3, F3, CLU, SERPINB2, TNFRSF18, HSPB1, IGF1, HSPA1A, SCG2). CCL2, CSF1, JUN, DDIT4* and *PPP1R15a* were also found between the highly expressed genes during lactation.

In D1 sample many DEGs were found in the statistically significant (*P* < 0.01) enriched GO terms associated with blood vessel development and morphogenesis (*GPI, PLXDCI, JUN, APOLD1, SEMAC3C, FGF1, FGF2, SCG2*); *GPI* and *JUN* were present also in the highly expressed gene list.

The WebGestalt KEGG/DAVID pathway enrichment analyses showed the glycolysis/gluconeogenesis and the metabolism of carbohydrates as specific for the D1 up-regulated genes (*GPI, TPI1, LDHA, SLC2A1, PGAM1, HK2, HK1, PGK1, GAPDH, ENO1*) while the cytokine-cytokine receptor interaction as the common pathway between the up (*IL1R2, CCL3, CCL2, CSF1, CCL5, CCL4, CCR9, ACVR1B, INHBA, CCL20, PPBP, TNFRSF18, IL12B, IL13RA1*) and down (*IL20RB, LIFR, CNTFR, IL7R, CD27, TGFB2*) regulated gene analysis (Additional file 6). Even the WebGestalt Pathway Commons analysis showed some pathway in common between D1 (*FN1, CCL2, NDRG1, SLC2A1, KAT5, RCAN1, IL13RA1, BHLHE40, CCL5, HSPA1A, DDIT4, JUN, FOSB, IGF1, TNFRSF18, EGLN3, ENO1, NT5E, SAP30, IGFBP3, TGIF1, MT2A, GAPDH, IGFBP1, LDHA, MMP1, ADM, MDK, FGF1, PGK1, CSF1, PPP1R15A, HK2, HK1, LBP, CLU, HSPB1*) and D2 (*PLCG1, PAOX, LEF1, CTLA4, CD4, GJA1, RET, NDRG2, MAPK13, COL4A1, TGFB2, ESR1, ETV1, FABP4, MYB*) up-regulated genes: integrin family cell surface interactions, glypican1 network and nectin.

Given the several biological functions and pathways identified through the analysis of gene expression in colostrum, we looked at the list of up regulated genes to search for predicted protein-protein interactions by using the STRING web resource. In the network view nodes are proteins while edges represent the predicted functional associations; the disconnected nodes were hidden (Additional file 8). Out of the 207 genes in the list, for 180 of them STRING found 97 protein interaction with a predicted confidence score > 0.7. The interaction enrichment analysis results showed that the network have significantly more interactions than expected.

### Functional enrichment analysis of DEGs in D2 sample

The down-regulated DEGs in colostrum and consequently up-regulated in mid lactation stage showed enrichment of the GO-BP terms associated with positive regulation of lymphocyte activation and anatomical structure morphogenesis. The most important GO-MF terms were associated with actin binding and molecular transducer activity; the most important GO-CC terms were associated with plasma membrane and cell surface (Additional files 6, 9).

Between DEGs many transporters were identified: solute carrier family 15 (H+/peptide transporter), member 2 (*SLC15A2*), solute carrier family 28 (concentrative nucleoside transporter), member 1 (*SLC28A1*), solute carrier family 5 (sodium/sugar co-transporter), member 9 (*SLC5A9*), solute carrier family 5 (sodium/glucose co-transporter), member 1 (*SLC5A1*).

The WebGestalt KEGG/DAVID pathway enrichment analyses showed the T cell receptor signaling pathway (*MAPK13, PLCG1, CTLA4, CD4, ICOS, CD28*), cell adhesion molecules (CAMs) (*ICOS, CTLA4, CD4, CD28*), Jak-STAT signaling pathway (*IL20RB, LIFR, CNTFR, IL7R*), VEGF signaling pathway (*PLCG1, MAPK13, PLA2G2C*) specific for the D2 up-regulated genes. WebGestalt KEGG identified two more terms in Fc epsilon RI signaling pathway and hematopoietic cell lineage. The WebGestalt Pathway Commons analysis showed statistically significant gene enrichment (*PLCG1, LEF1, CTLA4, CD4, GJA1, RET, NDRG2, MAPK13, TGFB2, ESR1, ETV1, FABP4, MYB*) of the class I PI3K signaling events mediated by Akt, signaling events mediated by Hepatocyte Growth factor Receptor (c-Met), EGFR-dependent Endothelial signaling events, IL5-mediated signaling events, IFN-gamma pathway (Additional file 6).

### Enrichment of oligosaccharides and lactose metabolism related genes

To identify the OS metabolism genes in goat milk we looked at the expression of 144 genes belonging to different functional glycosyltransferases commonly found in mammals, namely fucosyltransferases, galatosaminyl transferases, galactosyltransferases, sialyltransferases, mannosyltransferases, N-acetylglucosaminyl transferases. We included the glycosidases, fucose synthesis genes, sialic acid (SIA) synthesis genes, sugar transporters, and the genes for the lactose pathway in consideration that lactose L (Galb1-4Glc) forms the reducing end of milk oligosaccharides.

One hundred-ten genes out of 144 were expressed in the two milk samples with a minimum of 0.02 FPKM (*ST6GALNAC3*) in the D1 samples and a maximum of 60,453 FPKM (*LALBA*) in the D2 sample (Additional file 10). All genes of lactose synthesis, SIA synthesis genes, and fucose synthesis, were expressed in goat milk, whereas 34 genes belonging to the other categories were lacking, indicating a selective expression of some of these. Half of the genes showed a low expression (FPKM < 10) and half had medium expression value (>10 and <100 FPKM) while nine genes were expressed at higher levels, namely *LALBA, SLCA3*, GDP-mannose-4, 6-dehydratase (*GMSD*), NME/NM23 nucleoside diphosphate kinase 2 (*NME2*), *SLC2A1, B4GALT1*, UDP-GlcNAc:betaGal beta-1,3-N-acetylglucosaminyltransferase 2 (*B3GNT2*), N-acetylneuraminic acid synthase (sialic acid synthase) (*NANS)*, hexosaminidase subunit beta (*HEXB*).

**Fig. 4:**
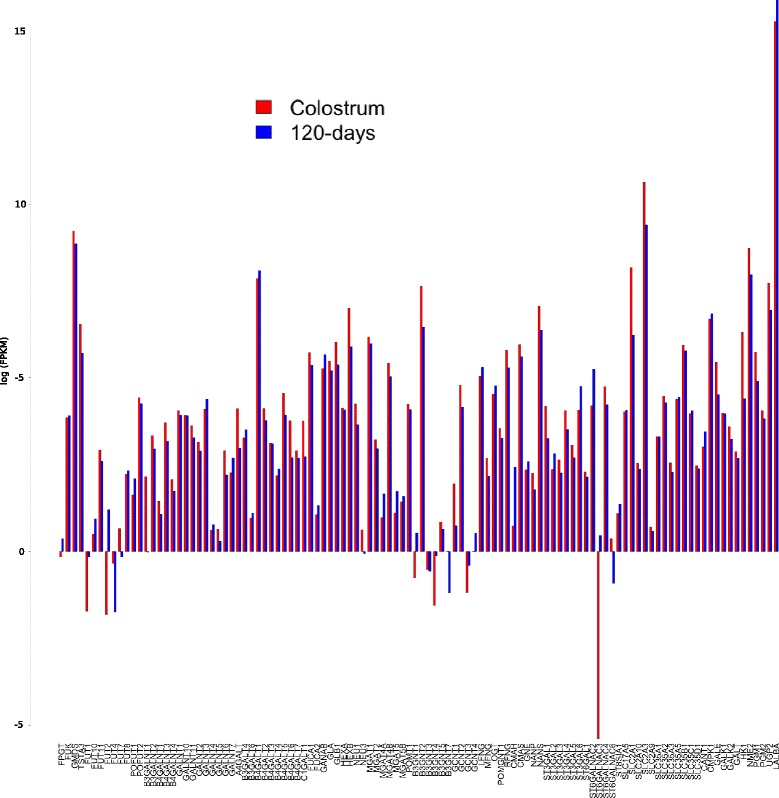
FPKM values of genes involved in goat oligosaccharides metabolism belonging to eleven functional categories

In Fig. 4 the log2 FPKM values for the expressed genes in D1 and D2 samples were graphically plotted. Most of the genes (64%) showed higher expression value in colostrum than in the later lactation stage, which can be better seen by plotting the log2 of the expression fold change (FC) (Additional file 11). The highest FC was for the *ST6GALNAC3* gene with a value of 69 in D2 sample even with a small FPKM expression value (1.38).

In general, almost all the genes coding for enzymes included in the lactose pathway showed an up-regulation in D1 stage; in particular, and increased *LALBA* and *B4GALT1* gene expression was revealed between D1 and D2.

Considering the other galactosyltransferase genes, the *B3GALT* family was expressed at lower level respect to *B4GALT* as found in bovine [33].

Among sialyltransferases genes, both α2,3-sialyltransferases and α2,6- sialyltransferases showed higher levels of expression in D1 than D2 sample except for *ST3GAL2, ST3GAL6, ST6GALNAC2, ST6GALNAC3, ST8SIA1. ST3GALT1, ST3GALT4, ST3GALT6, ST6GALNAC2, ST6GALNAC4* genes showed the highest expression and *ST3GAL1* showed a 4-fold change FPKM value with respect to *ST6GAL1*.

Among the *ST8SIA* family genes, only *ST8SIA1* was expressed in D1 and D2 samples.

Considering fucose synthesis, *GMSD* and *TSTA3* gene were highly expressed relative to *FPGT* and *FUK* both in D1 and D2 samples*.* Among fucosyltransferases, *FUT1, 2, 4, 7, 8, 10, 11* and *POFUT1* were found expressed even if at low levels, while *POFUT2* at medium level.

Among the N-acetylglucosaminyl transferase category, the *B3GNT2* gene was one of the higher expressed in D1 sample.

In the galactosaminyl transferase category, the *B3GALNT1* gene was expressed at higher level in D1 relative to D2 with a 4-fold change value.

A number of genes encoding proteins involved in complex N-glycan degradation were identified in goat milk, lysosomal sialidase (*NEU1*, *NEU3*), β-galactosidase *(GLB1*), β-hexosaminidase subunit α/β (*HEXA/HEXB*), α-L-fucosidase (*FUCA1, FUCA2*) able to catalyze the removal of Neu/NeuNAc, β-D-Gal, GlcNAc and Fuc, respectively, from the non-core structure of N-glycans. The *HEXB* was one of the most expressed genes overall and had high FPKM value with a two fold change ratio in D1 relative to D2 sample.

## Discussion

### RNA-seq mapping and gene expression statistics

In our experiment we choose to sequence the colostrum and 120 days samples at a resolution never used before in similar studies during lactation: i.e. more than 100 million mapped reads. Actually, forty millions have been considered sufficient to provide reliable measurement of a single transcript per cell and for the detection of major splice isoforms for abundant and moderately abundant transcripts [58]. However, greater sequencing depth allows more accurate assessment of the relative abundance of low-expression transcripts as well as the detection, by at least one read mapping, of a greater number of genes [59, 60]. In this work we wanted to explore as much as in detail the expressed genes in very different physiological stages, therefore we privileged having high sequencing depth and we chose to not pool samples and do not include biological replicates.

Considering the *de novo* assembly approach we compared our results with a similar study on goat mammary gland in which 98,864 transcripts and 51, 361 unigenes with a mean length of 1,219 bp were obtained [61]. Numbers are comparable, even if smaller, to those obtained by us. Moreover the authors used RNA pools of tissue at four life times and constructed a reference dataset for gene annotation without using a reference genome.

We compared our results with similar RNA-seq profiling in bovine milk somatic cells. In all these studies different experimental design, sampling, and sequencing depth were described but from a global point of view a media of 15,000-24,000 expressed genes have been identified [27, 28, 36]. In a previous work a threshold value of 0.2 RPKM was found for detectable gene expression in milk somatic cells [58]. If we consider the category of a more conservative FPKM value > 1, we get a number of co-expressed genes comparable to that found in bovine MSCs, here defined as those genes expressed at the FPKM threshold in both samples. In an analogous work in sheep a minor number of expressed genes was found, i.e. about 10,000 [29].

### Global milk transcriptome functional annotation and pathway analysis

Through the analysis of global milk transcriptome we noted that pathways involving nucleosides and ribonucleosides were enriched in colostrum. These compounds belong to the non-protein-nitrogen fraction, with biological role which is not limited to act as metabolites but to work as bioactive substances in the regulation of body functions, contributing to cell renewal or restoration [62]. Moreover nucleotide sugars act as donors for sugar residues in the glycosylation reactions that produce polysaccharides They are the glycosyl donors for glycosyltransferases and the precursors of glycoproteins, glycolipids and OS [63]. On the other hand, also the lysine degradation pathway was highly represented, in agreement with the findings of [64] suggesting that when Lys is in excess of requirements, the mammary gland appears to dispose of the extra supply via the oxidative mechanism. An increase in the catabolism of several amino acids was indicated in different studies on bovine mammary gland [65, 66], because amino acids could be an important pool of energy during lactation. The results of the BLAST2GO pipeline obtained in the present study confirm therefore the ability of the mammary gland to adapt different molecular functions according to the biological need of the animal.

### Top genes expressed in colostrum and mid lactation

Among the top expressed genes in colostrum and mid lactation, we detected *CSN2, LALBA, CSN1S1, CSN3 FTH1, SPP1, FASN a*nd *EEF1A1*, also identified by [57] among the top 20 expressed genes in the milk fat layer of mature human milk. The high expression of casein (*CN*) genes, all increasing in expression from D1 to D2 lactation stages, was not surprising because caseins, together with the whey proteins, represent the 90% of milk proteins fraction [67]. In this work, we found that *CSN2* and the whey protein α-lactalbumin (α-LA) coding gene (*LALBA*) have the highest expression values, as already detected by [61] in goat, [33] in cow and [507] in human.

The *PAEP* gene, also known as BLG, encodes the beta-lactoglobulin, the major whey protein in bovine, buffalo, goat, and horse milk [68], while *EEF1A1* high expression is justified by the considerable request of milk protein synthesis during lactation, being *EEF1A1* one of the most abundant protein synthesis factors [69, 70].

As for the *CTSD* (cathepsin D) gene, studies on rat mammary gland demonstrated that *CTSD* dependent activity is required to effect the cleavage of prolactin, the fundamental hormone for the function of the mammary gland, influencing also the expression of a number of genes as well the secretion of lipids and milk proteins [71–73].

The *FTH1* gene encodes for ferritin, and has been identified as an anti-apoptotic factor [74]. Ferritin is present in blood and extracellular fluids, including milk. In cows it was found that ferritin concentrations were higher in colostrum than in serum [75], in agreement with [57] who reported a higher ferritin genes expression in human colostrum with respect to mature milk. The expression of *M-SAA3.2* gene in the bovine mammary gland was reported by [76] who confirmed the role of this gene in host defense, likely in the neonate gastrointestinal tract. Moreover it has been suggested that *M-SAA3* represent one of the most promising candidates affecting milk protein and fat percentage of dairy cattle [27].

The *SPP1* gene, encoding for ospeopontin protein, is essential for mammary gland development, milk production, local mammary gland immunity and it has a significant role in the modulation of milk protein gene expression [77, 78]. The *FASN* gene is directly involved in *de novo* synthesis of most of the short and medium-chain fatty acids in milk and is essential for the development, functional competence, and maintenance of the lactating mammary gland [61, 79, 80]. *KRT19* gene expression has been associated with an intact cytoskeleton required for autophagic cell death in goat mammary gland [81]. The *GLYCAM1* gene encodes for a milk fat globule glycoprotein member of the mucin family. *GLYCAM1* mRNA was reported to be highly expressed during lactation in goat mammary gland [82], so suggesting a protective role of this gene.

High expression of the *RPS29* gene in goat mammary gland was already reported by [83] and in human mammary gland epithelium by [84].

### Functional annotation analysis of highly expressed genes during lactation

The finding of most statistically significant GO terms associated with translation, ribosomial biogenesis, rRNA processing is in agreement with the 25% of the highly expressed ribosomal protein genes. This is biologically meaningful because an increased translational requirement is needed to support caseins and other milk proteins synthesis. The gene encoding for the EEF2 translational elongation factor has been found expressed during goat lactation as previously reported in bovine [66]. However in ruminants mammary gland only a minor portion of the synthetic machinery appears to be used for production of milk proteins but for the biological needs of mammary tissue during lactation [85].

Many highly expressed genes during lactation were also associated to GO terms implicated in regulation of programmed cell death and cell proliferation relative terms. Mammary apoptosis was first demonstrated during tissue involution after lactation, but it has been detected also during lactation, in mammary tissue of lactating mice, goats and cattle [86, 87]. In bovine mammary tissue a high level of apoptosis was found in early lactation [88–90]. These authors speculated that their results were an effect of tissue edema and increased apoptotic leucocytes or that may be due to death of nonfunctional or senescent cells or to removal of excess capacity of newly synthesized and yet undifferentiated cells. In colostrum samples many different somatic cells type (leukocytes and macrophages) are present beyond milk epithelial cells, so we can suppose that both hypothesis could be corroborated by our results; on the other hand, we did not find, in the present trial, any of the most apoptotic markers (i.e. *Bax, Bcl-2, TGF-β1, CPP-32*, *Fas, CASP* genes) [90–92].

*UBB, UBA 52* and *RPS27A* genes represent three of the four ubiquitin genes found in mammals, expressing the main non-lysosomal route for intracellular protein degradation in eukaryotes. Both [93] and [28] found genes belonging to the ubiquitin-proteasome pathway expressed during early involution of mouse mammary gland and at peak lactation in bovine MSC, respectively. Recent findings also substantiate a pivotal role of the ubiquitin/proteasome pathway in the regulation of apoptosis [94, 95]. Also the NFκBIA gene, here found as highly expressed both in D1 and D2 samples, could have a role in the regulation of cell proliferation and programmed cell death because involved in activation of stressful situations requiring rapid reprogramming of gene expression [96]. It was reported to be expressed in bovine mammary gland during lactation by [90].

Heat shock protein genes (*HSPA5, HSPA6, HSPA8, HSP90AA1, HSP 70.1*), found between the highly expressed genes in the lactation, have a protective function, allowing cells to survive lethal conditions, and also interact with various components of the programmed cell death machinery; on the other hand, membrane bound HSPs mediate immunological functions [97, 98]. Our finding confirms what reported in literature about HSP functions and demonstrates how MSCs, in goat colostrum, can support a high level of protein synthesis act to operate in both immunity and apoptosis.

Another highly expressed GO category of genes included the nervous system and the regulation of transmission of nerve impulse, already reported by [65] in cow lactation given its role in milk ejection and control of blood flow.

Most of the highly expressed identified genes were specifically involved in carbohydrates metabolism. A previous study on goat mammary gland showed how glucose is oxidized about twice as rapidly as acetate and is essential for milk secretion and that the glucose uptake by the lactating gland was much greater than that required for milk lactose synthesis, suggesting that substantial amounts are oxidized under normal conditions, as in the isolated gland [99]. On the other hand the glucose transporter *SLC2A3* was highly expressed in both lactation stages, in agreement with the findings of [66] on cow lactation; these authors suggested that the high expression of genes involved in energy metabolism was likely related to the high-energy demand for milk synthesis and secretion [65].

### Differentially expressed genes between lactation stages

One of the major focuses of the present trial was to evaluate the differentially expressed genes between lactation stages, to assess and confirm the high nutritional value of goat colostrum and milk. Colostrum is a source of immunomodulatory molecules that positively influence the immune status of the neonate, including polymorphonuclear leukocytes and macrophages, T-lymphocytes, B-lymphocytes, plasma cells and epithelial cells [100, 101]. In the colostrum stage, the present study identified as up-regulated genes encoding carbohydrate transporter (*SLC2A1* and *SLC2A10*) and the nucleotide sugar transporter gene *SLC35E1.* Glucose transport across the plasma membranes of mammalian cells is carried out by two distinct processes employing the passive, facilitative, energy-independent glucose transporters GLUT, encoded by *SLC2A* genes, and the active sodium-dependent and energy-dependent glucose transporters SGLT, encoded by *SLC5A* genes. The member of the solute carrier family *SLC35* encode antiporters transporting nucleotide sugars pooled in the cytosol into the lumen of the Golgi and/or the ER in exchange for the corresponding nucleoside monophosphates. The transported nucleotide sugars are utilized as sugar donors by glycosyltransferases for the synthesis of sugar chains of glycoproteins, glycolipids and polysaccharides [102]. Currently, the SLC35 human family comprises 31 hydrophobic and homologous proteins, which are divided into 7 subfamilies, from SLC35A to SLC35G. Among the 31 SLC35 family members, only about one-third of them have been studied in detail. Little is known about the remaining members of the family, particularly the members of the SLC35E, SLC35F and SLC35G subfamilies and to date there are no known reports on the biological functions and the subcellular localizations of these proteins [103]. *SLC35E1* gene expression was found to be ubiquitous in various human adult and fetal tissues and cell lines with the highest value in mammary gland [104]. So our results confirm the expression of this gene in GMSC. Moreover we found the polysaccharides and glycosaminoglycan binding molecular function enriched in colostrum sample. This suggests a possible support to the oligosaccharides metabolism whose concentration has been reported to decrease from colostrum to late lactation stage in goat milk [105].

The finding of genes annotated in immune and defense terms in D1 sample is consistent with the general conception that milk after parturition provides passive protection for the neonate and has immunostimulatory capabilities. The chemokinesis and chemotaxis properties of goat milk toward different leukocyte populations were studied during lactation; mononuclear cells (MNCs) were the major cell found in colostrum and polymorphonuclear leukocytes (PMNs) the predominant cell type found during the late lactation stages (>weak 19) [106].

Many DEGs up-regulated in D1 were found in GO terms relative to either positive or negative regulation of cell proliferation and cell growth, as well as to apoptosis and programmed cell death. In the colostrum sample the protein encoded by chemokine (C-C Motif) Ligand 2 (*CCL2)* gene seems to have a positive effects on cell proliferation and a negative effects on apoptosis; this cytokine displays chemotactic activity for monocytes and basophils but not for neutrophils or eosinophils [107]. Among the genes involved in the positive regulation of cell proliferation, the fundamental role of the Colony Stimulating Factor 1 (*CSF1)* gene has to be underlined. Being the protein encoded by this gene a cytokine controlling production, differentiation, and function of macrophages, it promotes the release of proinflammatory chemokines, so playing an important role in innate immunity and in inflammatory processes [108]. Hence, we can argue that in colostrum cells the expression of both *CCL2* and *CSF1* genes could be involved in the recruitment of monocyte/macrophage to participate in immune responses, development, and tissue homeostasis and in monocyte recruitment in mammary gland tissue where collateral vessels are actively [109]. Furthermore the finding of DEGs in enriched GO terms associated with blood vessel development and morphogenesis agree with a study in bovine lactation [58] and confirm an important role for angiogenesis at the onset of goat lactation, while increasing blood flow across the mammary gland [110].

Of the common pathway of the up-regulated genes identified in the two lactation stages, integrins occupy an important role. Through integrins, the extra-cellular matrix influences growth, differentiation, and survival of mammary epithelial cells. Both in mouse [93] and bovine mammary gland [111] these proteins has been found expressed at different level during lactation and involution. In D1 sample enrichment of *IGF-1, IGFBP1* and *IGFBP3* genes was here revealed. The IGF system plays a pivotal role in mammary tissue homeostasis, regulating cell proliferation and differentiation during lactogenesis [112]; furthermore. *IGF-1* and *IGFBP3* were found expressed in bovine mammary gland at higher level during early lactation [89], while in goat mammary gland they have been found expressed during involution [113].

In the predicted protein interaction network the up-regulated genes in D1, obtained with STRING, the central role of the Jun Proto-Oncogene (*JUN)* is outlined by its multiple connections with other proteins. *JUN*, which was also one of the highly expressed genes, encodes a member of the activator protein-1 (*AP-1*) transcription factor family, a key component of many signal transduction pathways. AP-1 is composed of dimers of Jun (cJun, JunB, and JunD), Fos (cFos, FosB, Fra-1, and Fra-2), or other closely related factors such as ATF proteins. The Jun members homodimerize or heterodimerize with different Fos or ATF members, while Fos members only heterodimerize with different Jun members [114]. So the predicted *JUN* interaction with *FOSB* in the network has experimental biological relevance. Differential expression and activation of Jun and Fos members allow AP-1 complexes to control a wide variety of cellular functions. The AP-1 factor was indicated as a regulator of postnatal proliferation of mouse mammary epithelial cells [114] and of the early and late phases of mouse mammary gland involution [115]. The genes that are activated by FosB are unknown, but one excellent candidate is the oxytocin receptor gene which contains within its promoter an AP-1 site. A requirement for FosB in the nurturing response in mice was demonstrated [116], raising the hypothesis that FosB might be induced by oxytocin binding to receptors on preoptic area of the hypothalamus (POA) neurons, this leading to increased expression of the oxytocin receptor gene. Thus, by a feed-forward mechanism, POA neurons would express more oxytocin receptors and would therefore have enhanced sensitivity to oxytocin.

Some of *JUN* interactions could be relevant in cells physiology, as the interaction with the various *CCL* proteins and *IL12B*, which plays a role in cell proliferation/cell growth and immunity. The interaction with *FGF2/FGF1* could be explained by their inclusion in the same GO terms functional enrichment, the blood vessel development and morphogenesis. *JUN* is predict to link to *HSPB1*, which is in turn strongly associated to *HSPA1A/DNAJB1/BAG3*. All these genes are involved in cell maintenance and cellular assembly and organization. Small heat shock proteins (sHsp), of which *HSPB1* is a member, comprise the most widespread but also the most poorly conserved family of molecular chaperones and are involved in protecting cells from various unfavorable conditions, therefore playing a fundamental role in cell survival. At a molecular level, sHsp are involved in preventing aggregation of partially misfolded proteins and have been shown to intervene in modulating cell death pathways by interacting with various components of the cell death machinery upstream and downstream of the mitochondrial apoptotic events so preventing apoptosis in different lethal stress situations [117, 118]. At the bottom side of the network graph, the nine genes (*GAPDH, PGK1, TPI1, ENO1, LDHA*, *PGAM1, GPI, HK1, HK2*) linked together with many interaction edges belong to the glycolysis/gluconeogenesis KEGG pathway and carbohydrate metabolism biological process, which are two of the functional enrichment terms that significantly differentiate D1 from D2 lactation stage. The link between *JUN* and *GAPDH* derives from text-mining, because co-mentioned in a PubMed abstracts (*GAPDH* was used as gene reference gene).

Considering the up-regulated DEGs in D2 samples, some of these belong to the solute carrier family (*SLC*), coding for the SGLT family proteins. SGLT is a family of solute-linked carriers that contain sodium-coupled transporters for several nutrients. The Na +−electrochemical gradient provided by the Na +−K+ ATPase pump is utilized to transport substrates into cells against its concentration gradient. The co-transported substrates are sugars, inositol, proline, pantothenate, iodide, urea and other undetermined solutes [119]. In cattle, the glucose transporter SGLT1, encoded by *SLC5A1* gene, is responsible for the high-affinity, conservative uptake of most monosaccharides (glucose and galactose), except fructose; in human, SGLT4, encoded by *SLC5A9* gene, is an essential transporter for mannose, 1,5-anhydro-D-glucitol, and fructose [120]. In mammary gland epithelia, the protein encoded by the *SLC15A2* gene (PEPT2) is reported to be involved in the reuptake of small peptides accumulated from the hydrolysis of milk proteins [121]. The *SLC28A1* gene encodes for sodium-dependent, concentrative nucleoside transporter, pyrimidine- nucleoside preferring, epithelia localized, CNT1. We might conclude therefore that, both in D1 and D2 stages, sugar and nucleotide sugar transporters were differentially expressed to take account of the different physiological need of the mammary gland during lactation.

Within the more specific pathways of the up-regulated genes in D2 stage, the T cell receptor signaling, cell adhesion molecules, Jak-STAT signaling and VEGF signaling pathways must be mentioned.

In particular, the JAK-STAT signaling, which regulates many cellular processes, including innate and adaptive immune function, development, cell proliferation, differentiation, and apoptosis, has been found to be crucial in the mammary gland with STAT5 factor been important for controlling expression of milk protein genes in rodent [122]. However, in cattle and sheep mammary gland, the role of JaK2-STAT5 signaling pathway seemed to support a minor role for milk protein synthesis [29, 66]. In the present work we did not find *STAT5* between the highly expressed genes either in D1 or D2 samples but we found the transcription factor E74-like factor 5 (*ELF5*) expressed. This factor has been postulated to work as regulator of milk protein expression, encompassing STAT5 as a key player and its expression has been found increased during cow and sheep lactation [29, 66]. Therefore our findings allow to hypothesize a similar physiological role of this gene during lactation in goats.

In conclusion, the results obtained for the genes up-regulated in mature milk depict a scenario in which cells are ready to respond at external and immunological stimuli, to transduce signals inside cells and to orchestrate appropriate actions (e.g. cytokine gene transcription, JAK activation responsible for apoptosis execution) [123–126].

### Enrichment of oligosaccharides and lactose metabolism related genes

The synthesis of free OS and OS chain on glycoconjugates is mediated by the concerted action of many glycosyltransferases (GTs), glycosidases, sulfotransferase and accessory molecules; GTs typically catalyze the transfer of a monosaccharide from a nucleotide-sugar to a growing acceptor-OS attached to a carrier (in some cases directly to the carrier molecule). The OS product of one reaction is the substrate in the next reaction, and the array of glycosylation-related genes expressed in a particular cell is an important factor in the control of OS synthesis [20].

In this work we identified many expressed genes encoding for protein involved at different steps in the OS metabolism.

α-lactalbumin (*LALBA*) has important biological and functional properties and, in the mammary gland, interacts with β1,4-Galactosyltransferase (*B4GALT1*) to form the lactose synthase complex that produce lactose, the core unit from which all the other OS are synthesized [127, 128]. The increased *LALBA* expression that we found between D1 and D2 is consistent both with an increase of milk protein output and with lactose synthesis. The *B4GALT1* gene showed a high expression during goat lactation suggesting that the protein essential for the lactose synthase complex formation is ensured. Almost all the other genes coding for enzymes included in the lactose pathway showed an up-regulation in D1 stage similarly to what reported for human milk [57].

The *B4GALT1* encoded protein is one of the best-studied galactosyltransferase enzymes and has been identified on the plasma membrane, so that roles in cell-cell or cell-substrate recognition were hypothesized [129].

The genes implicated genes in sialylated OS metabolism were found expressed at medium values in our experiment. Considering the SIA synthesis genes [130], the *NANS* and the cytidine monophosphate N-acetylneuraminic acid synthetase (*CMAS*) were the most expressed, similarly to what found in bovine [33]. Goat milk has been reported to contain a high concentration of sialylated OS, specifically 3′sialyllactose (3′-SL), 6′sialyllactose (6′-SL); their biosynthesis starts from Gal in lactose with the addition of N-acetylneuraminic acid (NeuAc) or N-glycolylneuraminic acid (NeuGc), in α2-3 aor α2-6 linkages respectively. A detailed qualitative and quantitative characterization of goat colostrum oligosaccharides has been carried out by [131]. The authors identified 78 oligosaccharides, of which 40 (51.3%) were neutral non-fucosylated, 3 (3.8%) neutral fucosylated and 35 (44.9%) corresponded to sialylated (Neu5Ac/Neu5Gc) oligosaccharides. The predominant OS were sialyl-lactoses followed by Hex-HexNAc-Neu5Ac and Hex-HexNAc-Neu5Gc residues. The most abundant OS were galactosyl-lactoses.

The sialyltransferase enzymes *ST6GAL1* and *ST3GAL4* have been shown to be responsible for the production of 6′-SL and 3′SL in mouse milk respectively [132]. So we can suggest that both in goat *ST6GAL1* could act in the biosynthesis of 6′SL and *ST3GAL4* plus *ST3GAL1* and *ST3GAL6* in the biosynthesis of 3′SL. Our results agree with the literature both because higher OS concentrations have been reported in colostrum than in late lactation in different animal species and given that some oligosaccharide profile analyses indicated how 3′-sialylactose is more abundant than the 6′-sialylactose in colostrum and late lactation goat milk [105, 133].

*ST3GAL5* encodes the GM3 synthase responsible for the biosynthesis of ganglio-series gangliosides that are sialic acid-bearing glycosphingolipids, the major glycans in nerve cells. It was recently demonstrated that ST3Gal5 catalyzes GM4 synthesis in vivo [134–136] suggesting that the sialic acid moieties of gangliosides and glycoproteins in the brain frontal cortex probably participate in a variety of cellular events including the formation of memory and learning. It was shown that it may be feasible to use sialyllactose, separated from cheese whey or colostrum, as a functional food for brain activation [137].

The protein encoded by one of the higher expressed gene in D1 sample (*B3GNT2*) synthesizes a unique structure known as poly-N-acetyllactosamine (polyLacNAc) a linear carbohydrate polymer composed of alternating N-acetylglucosamine and galactose residues. Recently, results obtained with knockout B3gnt2−/− mice strains showed that hyperactivation of lymphocytes occurred due to a lack of polylactosamine on receptor molecules, indicating that polylactosamine has an important role in immunological biofunctions [138, 139]. The analysis of gene expression profiles in human monocytes, dendritic cells and macrophages found expressed *B3GNT2* suggesting that monocytes synthesize N-glycans carrying di, tri, and tetra-antennary structures, possibly elongated by poly-N-acetyllactosamine chains. This agrees with our results of genes annotated in immune and defense terms in D1 somatic cell and with the finding that mononuclear cells (MNCs) are the major cells found in colostrum [106].

The expressed *B3GALNT1* gene encodes for the globoside synthase an enzyme that catalyze the addition of N-acetylgalactosamine to globotriaosylceramide (Gb3) to form globoside (Gb4), a globo-series glycosphingolipid (GSL) member [140]. Because of the relationship to many human pathogens and diseases the interest in glycolipid-based blood group antigens remains high. Moreover milk glycosphingolipids have been reported to participate in the newborn’s defense against pathogens. Ovine milk has been found to have a high content of complex neutral GSL, such as Gb3 and Gb4, which are very abundant in human milk but not in bovine milk and which are able to bind to some *E. coli* strains to prevent infection [141].

In our samples many genes both for fucose synthesis and fucosyltransferase were found expressed. In contrast to human milk, the content of fucosylated oligosaccharides is rather low in the milk of domestic animal species [18]. In goat colostrum 2′FL was found by [142], while in [131] 2′-FL plus fucosyl-lactosamine and lacto-N- fuco-pentaose were detected even if at low abundance. During fucosylation, activated fucose is transferred to various lactosamine acceptors by specific fucosyltransferases (Fuc-T) encoded by the fucosyltransferase gene (*FUT*). The biosynthesis of GDP-L-fucose consists of two cytosolic pathways. The constitutively active *de novo* pathway involves two enzymes (GMD, encoded by *GMSD* gene and FX, encoded by *TSTA3* gene). In the alternative salvage metabolism, that utilizes L-Fuc obtained from extracellular sources or from intracellular degradation of glycoproteins and glycolipids, L-fucokinase (*FUK* gene) and GDP-L-fucose pyrophosphorylase (*FPGT* gene) are involved. After synthesis in the cytosol, GDP-L-Fuc is translocated into the Golgi apparatus via a specific GDP-fucose transporter encoded by gene *FUCT1/SLC35C1* [143, 144]. Because in D1 and D2 samples *GMSD* and *TSTA3* gene were highly expressed relative to *FPGT* and *FUK* we suggesting that in GMSC the *de novo* pathway is preferentially activated and that a high amount of fucose is synthesized in cytoplasm ready to be imported in Golgi apparatus for fucosylation. Among the fucosyltransferases, *POFUT2* gene was the highest expressed in D1 and D2 samples. The encoded soluble enzymes is localized in the endoplasmic reticulum (ER) and its ability to distinguish between folded and unfolded structures has led to the proposal that POFUT2 function as chaperones or in the folding and secretion of their target proteins [145].

Among glycosidase genes, the *HEXB* gene has been found highly expressed in both samples with a two fold change ratio in D1 relative to D2 sample. Two major lysosomal isoenzymes exist in human tissues, which are the products of the assembly of two subunits, α and β, encoded by the homologous genes *HEXA* and *HEXB*. The two isoenzymes Hex A (αβ) and Hex B (ββ) differ in their substrate specificity. The active site of the β-subunit hydrolyzes uncharged substrates, whereas the α-subunit, in addition, cleaves negatively charged substrates [146]. The greater expression of *HEXB* could be associated with the presence of higher percentage of neutral OS in goat milk. In bovine MSCs higher expression values for *HEXA* gene were found [33]. Moreover because lysosomal hexosaminidases release terminal β-glycosidically linked N-acetylglucosamine and N-acetylgalactosamineresidues from a number of glycoconjugates [147] we can speculate that the higher oligosaccharides concentration in colostrum could be due both to glycan degradation activities and glycosyltransferase activities.

Considering neuraminidase genes, *NEU2* and *NEU4* were not found expressed, as in bovine [33].

All the investigated sugar transporter genes were found expressed in goat milk, except *SLC2A8* and *SLC35A4.* These last two genes, contrary to goat, were present in bovine mammary gland and somatic cells [33, 66]. We already discussed *SLC2A1, SLC2A10* and *SLC2A3* as DEG and highly expressed genes respectively. Among the nucleotide sugars transporters, *SLC35A2, SLC35A5, SLC35B1* and *SLC35C1* were expressed at higher levels. Both the CMP-Sialic Acid Transporter (*SLC35A1*) [148] and GDP-Fucose Transporter 1 (*SLC35C1*) [149] were found expressed at low and medium levels, respectively. *SLC17A5* (also known as AST), encode a protein (sialin) with a sialic acid transport function whose mutations are the primary cause of lysosomal sialic acid storage diseases [150].

## Conclusion

Transcriptional profiling is a powerful approach for identification of genes globally and functionally expressed in various tissue including mammary gland and milk. In this work we characterized the goat milk somatic cells transcriptome at two lactation stages by using RNA-seq and by integrating two bioinformatic methodologies. We looked at the gene expression, using software applications that integrate functional enrichment analysis and statistical analysis of large set of genes. We identified distinct genes and pathway underlying the differences between colostrum and 120 days milk. Finally, for the first time, a study of glycosylation-related genes was carried out in goat milk to get a picture of genes possibly implicated in oligosaccharides biosynthesis.

## Additional files

Additional file 1: Cufflink/Cuffmerge transcriptome in D1 and D2 lactation samples. Information on gene annotation, Gene Ontology terms and Kegg pathways are included. (XLSX 5997 kb)
Additional file 2: Enriched Gene Ontology term analysis in D1 and D2 lactation samples. (XLSX 68 kb)
Additional file 3: Enriched Kegg pathway analysis in D1 and D2 lactation samples. (XLSX 98 kb)
Additional file 4: DAVID Functional Annotations and Clustering for highly expressed genes in D1 and D2 lactation samples. (XLS 154 kb)
Additional file 5: Differentially Expressed Genes (DEGs) between D1 and D2 lactation samples. (XLS 89 kb)
Additional file 6: DAVID Functional Annotations and Clustering for up-regulated and down-regulated genes in D1 sample. The most significant enriched GO categories under Biological Process, Molecular Function, and Cellular Component are included in a summary sheet. (XLSX 111 kb)
Additional file 7: Directed Acyclic Graphs (DAGs) for the Biological Process, Molecular Function and Cellular Component categories of up-regulated genes in D1 sample. Each node shows the number of genes in the category and the adjusted *p*-value indicating the significance of enrichment. GO categories in red are the enriched GO categories while the black ones are their non-enriched parents. GO categories in the top 10 that also have a *p*-value < 0.05 are colored in red. (PDF 146 kb)
Additional file 8: Network view of the up-regulated gene list in D1. In this view the network nodes are proteins and the edges represent the predicted functional associations. The edges are draw according to the view settings. In evidence mode, an edge may be drawn with up to 7 differently colored lines that represent the existence of the seven types of evidence used in predicting the associations. The fusion view shows the individual gene fusion events per species; the neighborhood view shows runs of genes that occur repeatedly in close neighborhood in (prokaryotic) genomes; the occurrence view shows the presence or absence of linked proteins across species; the experiments view shows a list of significant protein interaction datasets, gathered from other protein-protein interaction databases; the text mining view shows a list of significant protein interaction groups, extracted from the abstracts of scientific literature; the database view shows a list of significant protein interaction groups, gathered from curated databases; the coexpression view shows the genes that are co-expressed in the same or in other species (transferred by homology). (PDF 4384 kb)
Additional file 9: Directed Acyclic Graphs (DAGs) for the Biological Process, Molecular Function and Cellular Component categories of down-regulated genes in D2 sample. Each node shows the number of genes in the category and the adjusted *p*-value indicating the significance of enrichment. GO categories in red are the enriched GO categories while the black ones are their non-enriched parents. GO categories in the top 10 that also have a *p*-value < 0.05 are colored in red. GO categories in the top 10 that have a *p*-value > 0.05 are colored brown, and the black ones are the parents of the top 10 categories. (PDF 151 kb)
Additional file 10: List of the 110 genes belonging to eleven functional oligosaccharides metabolism categories with FPKM expression in D1 and D2 samples. (XLSX 17 kb)
Additional file 11: Graphical representation of the log2 (D1/D2 FPKM). (PDF 109 kb)

## Abbreviations

DEG: Differentially expressed gene
GO-BP: Gene ontology biological processes
GO-CC: Gene ontology cellular component
GO-MF: Gene ontology molecular function
