# Supplementary figures and images for: RNA-Sequencing for profiling goat milk transcriptome in colostrum and mature milk
Source: BMC Vet Res. 2016 Nov 25;12:264. doi: 10.1186/s12917-016-0881-7 (PMC5123407; doi:10.1186/s12917-016-0881-7)

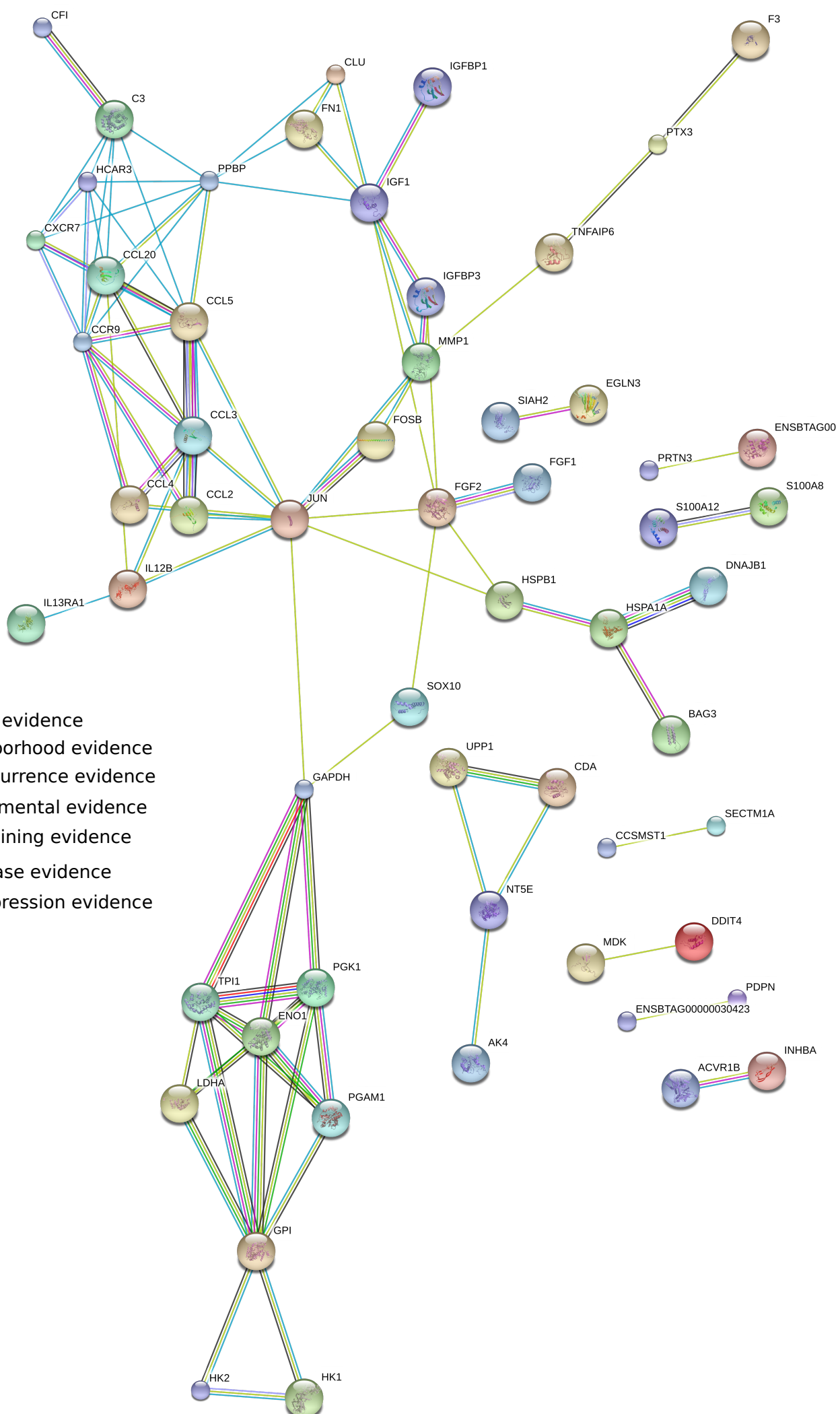

Supplement: Additional file 8: — Network view of the up-regulated gene list in D1. In this view the network nodes are proteins and the edges represent the predicted functional associations. The edges are draw according to the view settings. In evidence mode, an edge may be drawn with up to 7 differently colored lines that represent the existence of the seven types of evidence used in predicting the associations. The fusion view shows the individual gene fusion events per species; the neighborhood view shows runs of genes that occur repeatedly in close neighborhood in (prokaryotic) genomes; the occurrence view shows the presence or absence of linked proteins across species; the experiments view shows a list of significant protein interaction datasets, gathered from other protein-protein interaction databases; the text mining view shows a list of significant protein interaction groups, extracted from the abstracts of scientific literature; the database view shows a list of significant protein interaction groups, gathered from curated databases; the coexpression view shows the genes that are co-expressed in the same or in other species (transferred by homology). (PDF 4384 kb) [file 12917_2016_881_MOESM8_ESM.pdf]

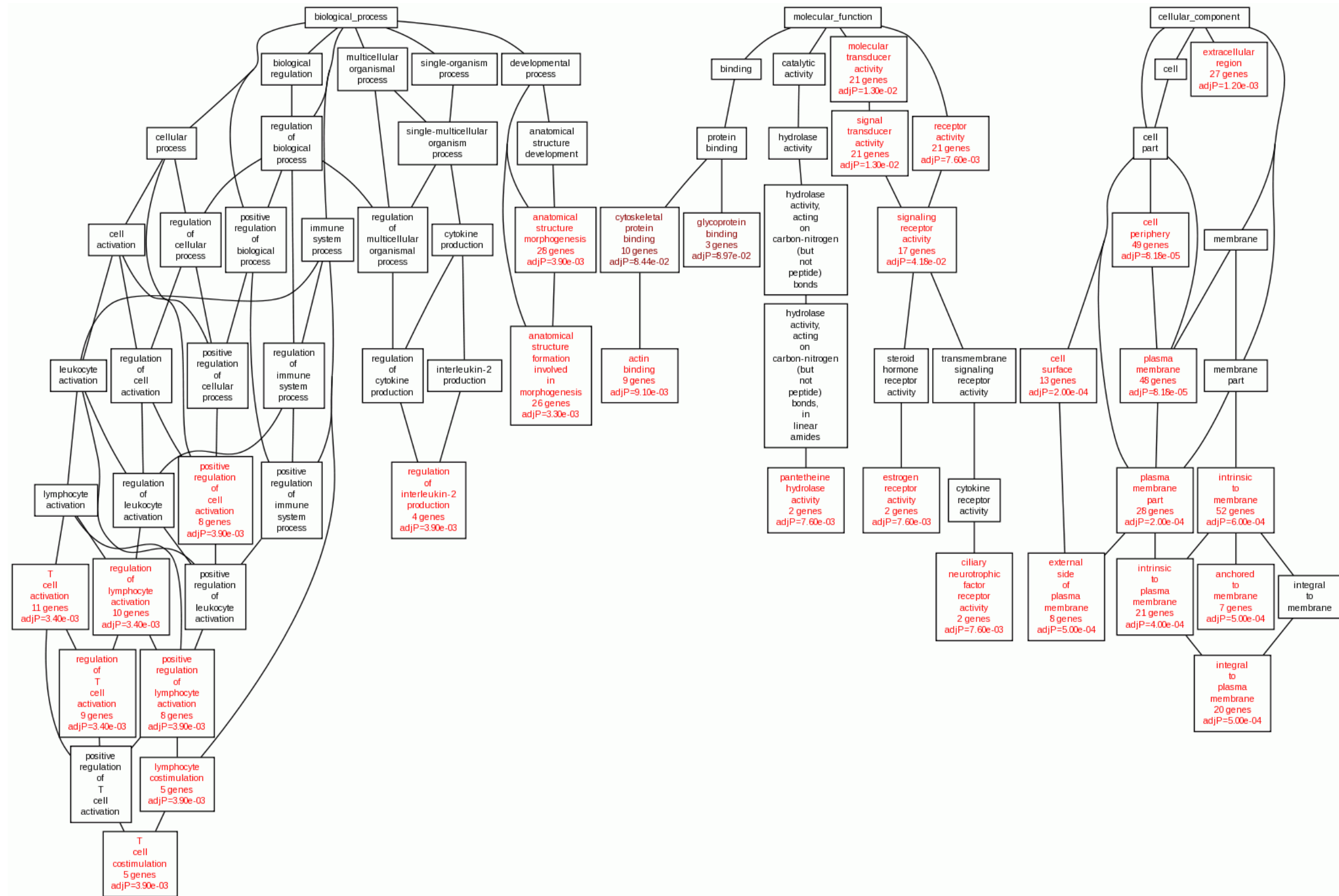

Supplement: Additional file 9: — Directed Acyclic Graphs (DAGs) for the Biological Process, Molecular Function and Cellular Component categories of down-regulated genes in D2 sample. Each node shows the number of genes in the category and the adjusted p-value indicating the significance of enrichment. GO categories in red are the enriched GO categories while the black ones are their non-enriched parents. GO categories in the top 10 that also have a p-value < 0.05 are colored in red. GO categories in the top 10 that have a p-value > 0.05 are colored brown, and the black ones are the parents of the top 10 categories. (PDF 151 kb) [file 12917_2016_881_MOESM9_ESM.pdf]

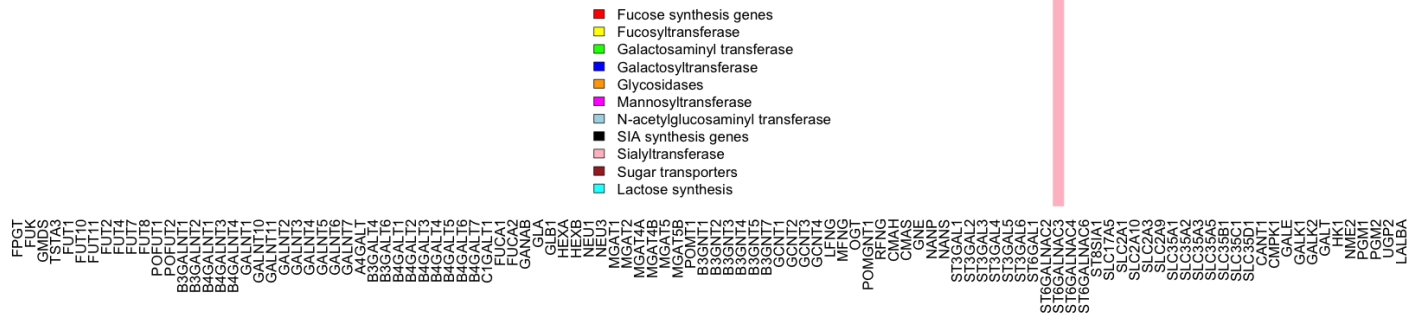

Supplement: Additional file 11: — Graphical representation of the log2 (D1/D2 FPKM). (PDF 109 kb) [file 12917_2016_881_MOESM11_ESM.pdf]
