# Supplementary material for: RNA-Sequencing for profiling goat milk transcriptome in colostrum and mature milk
Source: BMC Vet Res. 2016 Nov 25;12:264. doi: 10.1186/s12917-016-0881-7 (PMC5123407; doi:10.1186/s12917-016-0881-7)
Supplement: Additional file 7: — Directed Acyclic Graphs (DAGs) for the Biological Process, Molecular Function and Cellular Component categories of up-regulated genes in D1 sample. Each node shows the number of genes in the category and the adjusted p-value indicating the significance of enrichment. GO categories in red are the enriched GO categories while the black ones are their non-enriched parents. GO categories in the top 10 that also have a p-value < 0.05 are colored in red. (PDF 146 kb) [file 12917_2016_881_MOESM7_ESM.pdf]

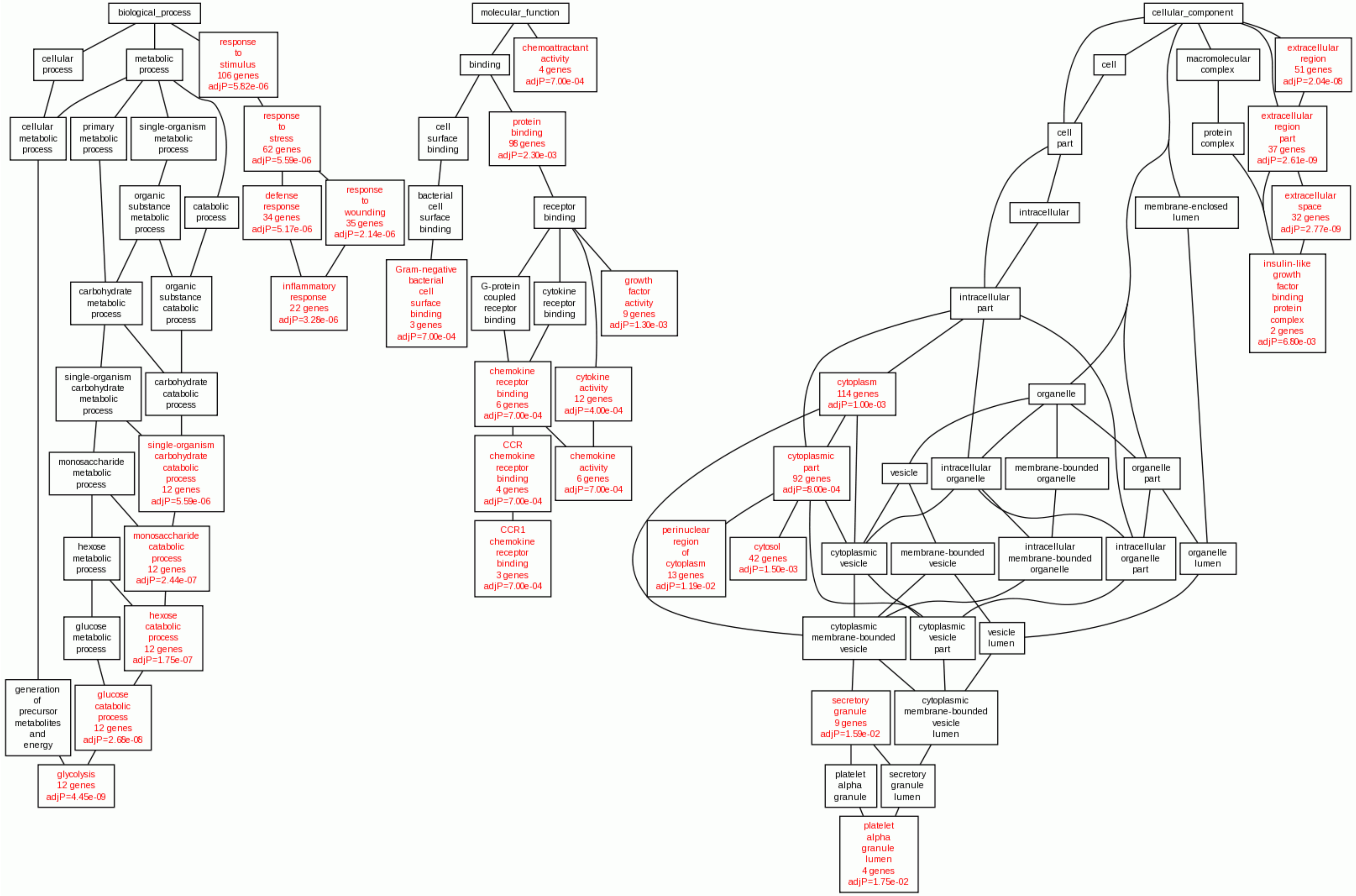

biological_process
cellular process
metabolic process
response to stimulus 106 genes adjP=5.82e-06
cellular metabolic process
primary metabolic process
single-organism metabolic process
response to stress 62 genes adjP=5.59e-06
organic substance metabolic process
catabolic process
defense response 34 genes adjP=5.17e-06
response to wounding 35 genes adjP=2.14e-06
carbohydrate metabolic process
organic substance catabolic process
inflammatory response 22 genes adjP=3.28e-06
single-organism carbohydrate metabolic process
carbohydrate catabolic process
monosaccharide metabolic process
single-organism carbohydrate catabolic process 12 genes adjP=5.59e-06
hexose metabolic process
monosaccharide catabolic process 12 genes adjP=2.44e-07
glucose metabolic process
hexose catabolic process 12 genes adjP=1.75e-07
generation of precursor metabolites and energy
glucose catabolic process 12 genes adjP=2.68e-08
glycolysis 12 genes adjP=4.45e-09
molecular_function
binding
chemoattractant activity 4 genes adjP=7.00e-04
cell surface binding
protein binding 98 genes adjP=2.30e-03
bacterial cell surface binding
receptor binding
Gram-negative bacterial cell surface binding 3 genes adjP=7.00e-04
G-protein coupled receptor binding
cytokine receptor binding
growth factor activity 9 genes adjP=1.30e-03
chemokine receptor binding 6 genes adjP=7.00e-04
cytokine activity 12 genes adjP=4.00e-04
CCR chemokine receptor binding 4 genes adjP=7.00e-04
chemokine activity 6 genes adjP=7.00e-04
CCR1 chemokine receptor binding 3 genes adjP=7.00e-04
cellular_component
cell
macromolecular complex
extracellular region 51 genes adjP=2.04e-08
cell part
protein complex
extracellular region part 37 genes adjP=2.61e-09
intracellular
membrane-enclosed lumen
extracellular space 32 genes adjP=2.77e-09
insulin-like growth factor binding protein complex 2 genes adjP=6.80e-03
intracellular part
cytoplasm 114 genes adjP=1.00e-03
organelle
cytoplasmic part 92 genes adjP=8.00e-04
vesicle
intracellular organelle
membrane-bounded organelle
organelle part
perinuclear region of cytoplasm 13 genes adjP=1.19e-02
cytosol 42 genes adjP=1.50e-03
cytoplasmic vesicle
membrane-bounded vesicle
intracellular membrane-bounded organelle
intracellular organelle part
organelle lumen
cytoplasmic membrane-bounded vesicle
cytoplasmic vesicle part
vesicle lumen
secretory granule 9 genes adjP=1.59e-02
cytoplasmic membrane-bounded vesicle lumen
platelet alpha granule
secretory granule lumen
platelet alpha granule lumen 4 genes adjP=1.75e-02
